# Global proteomic analyses of human cytotrophoblast differentiation/invasion

**DOI:** 10.1242/dev.199561

**Published:** 2021-07-01

**Authors:** Hao Chen, Katherine E. Williams, Elaine Y. Kwan, Mirhan Kapidzic, Kenisha A. Puckett, Rayyan K. Aburajab, Joshua F. Robinson, Susan J. Fisher

**Affiliations:** 1 Center for Reproductive Sciences, University of California, San Francisco, CA 94143, USA; 2 Department of Obstetrics, Gynecology, and Reproductive Sciences, University of California, San Francisco, CA 94143, USA; 3 Eli and Edythe Broad Center for Regeneration Medicine and Stem Cell Research, University of California, San Francisco, CA 94143, USA; 4 Sandler-Moore Mass Spectrometry Core Facility, University of California, San Francisco, CA 94143, USA; 5 Division of Maternal Fetal Medicine, University of California, San Francisco, CA 94143, USA; 6 Department of Anatomy, University of California, San Francisco, CA 94143, USA; 7 Human Embryonic Stem Cell Program, University of California, San Francisco, CA 94143, USA

**Keywords:** Human, Placenta, Cytotrophoblast, Proteomics, SWATH-MS

## Abstract

During human pregnancy, cytotrophoblasts (CTBs) from the placenta differentiate into specialized subpopulations that play crucial roles in proper fetal growth and development. A subset of these CTBs differentiate along an invasive pathway, penetrating the decidua and anchoring the placenta to the uterus. A crucial hurdle in pregnancy is the ability of these cells to migrate, invade and remodel spiral arteries, ensuring adequate blood flow to nourish the developing fetus. Although advances continue in describing the molecular features regulating the differentiation of these cells, assessment of their global proteomic changes at mid-gestation remain undefined. Here, using sequential window acquisition of all theoretical fragment-ion spectra (SWATH), which is a data-independent acquisition strategy, we characterized the protein repertoire of second trimester human CTBs during their differentiation towards an invasive phenotype. This mass spectrometry-based approach allowed identification of 3026 proteins across four culture time points corresponding to sequential stages of differentiation, confirming the expression dynamics of established molecules and offering new information into other pathways involved. The availability of a SWATH CTB global spectral library serves as a beneficial resource for hypothesis generation and as a foundation for further understanding CTB differentiation dynamics.

## Abstract (precis)

**Summary:** The generation of a publicly available SWATH-MS global spectral library of human second-trimester primary villous cytotrophoblasts differentiating along an invasive pathway.

## INTRODUCTION

The placenta is a transient organ that establishes and mediates maternal-fetal interactions during pregnancy. The creation of this interface depends on the proper development and function of the placenta's chorionic villi (Maltepe and Fisher, 2015). Throughout pregnancy, placental villi undergo extensive remodeling and branching, maturing into floating or anchoring villi. Most of the placental surface comprises floating villi that are in direct contact with, and suspended in, maternal blood. There, the exchange of nutrient, waste and gas is facilitated between the maternal and fetal compartments (Turco and Moffett, 2019). This is accomplished through the outermost epithelial layer of the villi, a multinuclear layer composed of syncitotrophoblasts (STBs) that is generated via fusion of the underlying cytotrophoblast (CTB) progenitors. In anchoring villi, these progenitors aggregate into cell columns that attach to the decidua. Invasive CTBs emanate from the cell columns and migrate deeply into the uterine wall. During this process, these extravillous trophoblasts remodel spiral arteries, which diverts blood flow to the placenta (Red-Horse et al., 2004). Proper regulation of CTB differentiation/invasion is crucial for successful pregnancy, and perturbations in this process are associated with several complications such as preeclampsia (PE) (Fisher, 2015), preterm labor (Romero et al., 2014) or placenta accreta spectrum (Jauniaux et al., 2018; McNally et al., 2020).

Given the unique structure and function of the human placenta (versus animal models), and the difficulty of *in utero* studies, primary human CTB models have been crucial for advancing our knowledge of how these cells function at a molecular level (Knöfler et al., 2019). Work from our group and others has revealed numerous components of the CTB repertoire that program differentiation/invasion, especially the vasculogenic component of this process. However, achieving a comprehensive global understanding of the dynamic changes that occur has yet to be realized. We previously characterized global gene expression changes in primary CTB cultures under conditions that promote acquisition of an invasive phenotype (Robinson et al., 2017). Numerous factors, however, can impact translational efficiency from transcript to protein (Tian et al., 2004). As a result, proteomic studies provide complementary biological insights as they carry out the majority of the cell's functions.

Mass spectrometry (MS)-based methods have developed rapidly. One such approach, Sequential Window Acquisition of All Theoretical spectra (SWATH), has emerged as a comprehensive, highly reproducible data acquisition workflow for proteomic studies (Collins et al., 2017). Additionally, this approach has the added advantage of enabling relative quantification without introducing a chemical labeling step. In SWATH-MS, spectra are recorded using an unbiased, data-independent acquisition (DIA) based on preselected mass range windows (‘swaths’). The results are then queried against a previously generated spectral library containing the spectrometric coordinates of proteins and peptides of interest (Ludwig et al., 2018). These ion libraries are a unique publicly available resource to which additional DIA data can be added over time, growing the repository and increasing its usefulness.

Here, we report the creation of a SWATH spectral library generated from a second-trimester human CTB culture model of differentiation/invasion that enabled the measurement of over 40,000 peptides from over 3000 proteins. We demonstrate the utility of this dataset by quantifying 3026 proteins across four culture time points. The dynamics of numerous molecules confirmed known expression patterns and revealed unique identification of molecules that could play roles in differentiation/invasion. Our study is the first reference SWATH library of second-trimester CTBs, as well as the first comprehensive description of the CTB proteome during differentiation towards an invasive phenotype.

## RESULTS

### Global proteomic analysis of human CTB differentiation

**Fig. 1.**
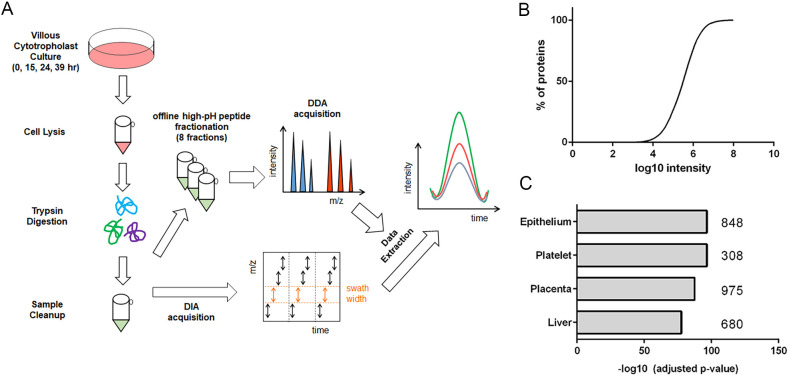
**Global proteomic profiling of cytotrophoblast differentiation/invasion.** (A) Simplified workflow schematic of cytotrophoblast proteomic analysis using SWATH-MS. (B) Distribution of relative protein abundances, spanning four orders of magnitude. (C) Tissue enrichment analysis of annotated proteins from the dataset. *n*=4 biological replicates.

A simplified workflow is diagrammed in Fig. 1A. To profile CTB proteome dynamics during differentiation towards an invasive phenotype, primary human CTBs were isolated and purified from second trimester placentas (gestational ages 20 to 24 weeks; *n*=4) and grown in serum-free medium (Hunkapiller and Fisher, 2008) (Fig. S1). Cells were harvested at 0, 15, 24 and 39 h, a range of time points corresponding with the acquisition of an invasive phenotype in cultured second trimester CTBs (Damsky et al., 1994). For quantitative proteomic analyses using SWATH-MS, lysates were trypsin digested and a pooled sample was fractionated and analyzed by LC-MS/MS to create an ion library, which was annotated. Overall, 3026 proteins were quantified across all time points with relative protein abundances spanning over four orders of magnitude (Fig. 1B). A heatmap of Pearson scores among individual biological replicates illustrated a high degree of correlation (Fig. S2A). The coefficient of variation among samples for each time point was low, with a median of ∼0.3 (Fig. S2B). Tissue enrichment analysis using the *Homo sapiens* proteome as background showed that a large proportion of the CTB proteome included placental proteins (975) and, in keeping with their ectodermal origin, proteins (848) that are typically found in epithelia (Fig. 1C). Overlap between the CTB proteome and that of platelets and liver is consistent with the anti-coagulant and hepatic-like functions of these cells, respectively. Gene Ontology (GO) Enrichment analysis of the CTB proteome showed an abundance of proteins associated with localization (615) and transport (689) processes, with an overrepresentation of RNA binding (664) and cell-cell adhesion (228) (Fig. S2C). Additionally, with respect to cellular compartments, over one-third of the proteins had exosomal or vesicular annotations (1137).

### Protein dynamics during human CTB differentiation/invasion *in vitro*

To identify differentially expressed (DE) proteins during CTB differentiation (0-39 h), we applied an integrated linear model to our dataset (LIMMA) (Ritchie et al., 2015). In total, we identified 477 proteins that had significant changes in abundance relative to 0 h (*P*<0.05).

**Fig. 2.**
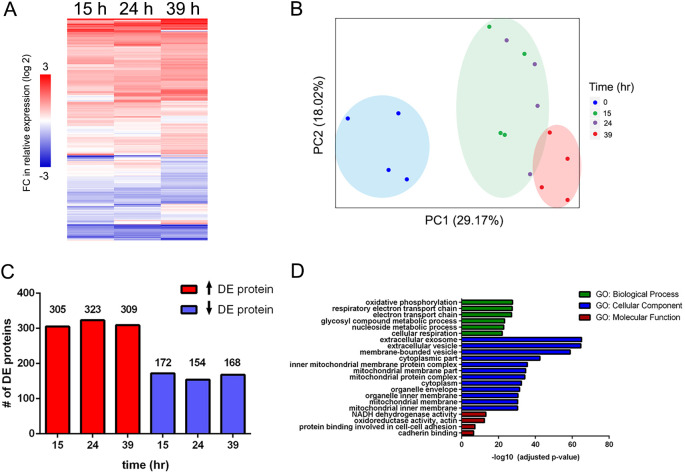
**Differentially expressed protein changes during cytotrophoblast differentiation/invasion.** (A) Unsupervised hierarchical clustering of 477 differentially expressed (DE) proteins relative to 0 h. (B) Principal component analysis of DE proteins by biological replicate (*n*=4). (C) Distribution of DE protein expression changes at each time point relative to 0 h. (D) Gene ontology (GO) enrichment analysis of DE proteins. The most enriched categories from the three GO terms (biological process, cellular component and molecular function) were primarily matched to respiratory/metabolic processes, exosomal/vesicular components and metabolic/adhesive functions, respectively (Fisher's exact test; *P*<0.01, Benjamini-Hochberg corrected).

**Fig. 3.**
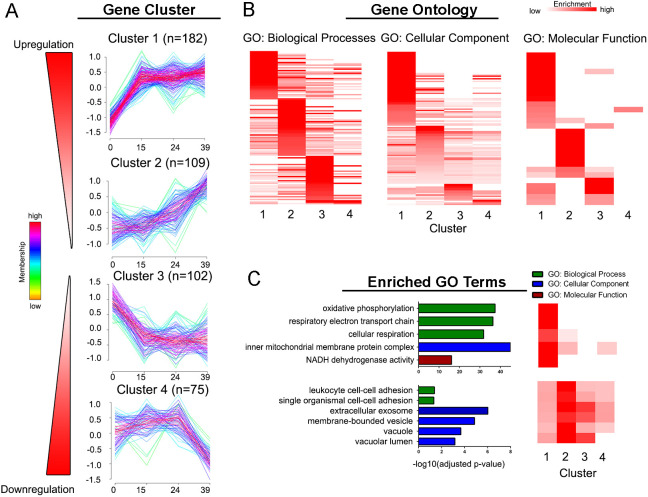
**Dynamics of differentially expressed proteins during cytotrophoblast differentiation/invasion.** (A) Unsupervised fuzzy clustering of 477 differentially expressed (DE) proteins identified four clusters with distinct expression profiles. *n*=number of proteins assigned to each cluster. (B) Heatmap of gene ontology (GO) enrichment analysis of each cluster. GO terms overrepresented in each cluster (Fisher's exact test; *P*<0.05, unadjusted) were visualized by fold enrichment as a qualitative comparison to functionalities in other clusters. (C) Representative GO terms enriched in clusters 1 and 2 (Fisher's exact test; *P*<0.05, Benjamini-Hochberg corrected) were visualized for biological processes, cellular component or molecular function (MF).

In general, a majority of the DE proteins showed upregulation (Fig. 2A). The magnitude of expression also increased with time, with ∼38% (181/477) of DE proteins at 39 h showing more than a twofold change (log_2_scale>|1|) (Fig. 3A-C). Principal components analysis (PCA) of the DE proteins showed their ability to distinctly cluster each sample according to culture time, with the lowest variance between 15 and 24 h, and the largest variance between 0 and 39 h (Fig. 2B). Fig. 2C shows the number of proteins whose abundances changed at each time point relative to CTBs prior to culture (0 h). With regard to functional analyses, DE proteins were most significantly enriched in metabolic and adhesive processes. Notably, nearly half of the DE proteins (48%) had exosomal or vesicular annotations, highlighting the importance of extracellular vesicles to CTB differentiation, including possible autocrine and/or paracrine roles (Fig. 2D).

Of the proteins that were not significantly changing, enrichment analyses revealed a continued overrepresentation of proteasomal, ribosomal and transport processes, suggesting that the levels of proteins associated with many fundamental cellular processes remained relatively stable during CTB differentiation (Fig. 2D).

### Patterns of temporal shifts in the CTB proteome

We performed an unsupervised fuzzy clustering of the DE proteins to stratify their temporal dynamics. Four distinct clusters were identified (Tibshirani et al., 2001): two upregulated (1 and 2) and two downregulated (3 and 4) (Fig. 3A). Many proteins in each cluster showed shared functionalities and cellular localization (Fig. 3B), with clusters 1 and 2 possessing significantly distinct biological processes at particular stages of differentiation (Fig. 3C).

Cluster 1 (1 and 2 proteins) had a pattern of rapid upregulation after 0 h followed by sustained expression to 39 h. This included proteins, e.g. HLA-G, that our group have shown are upregulated in the early stage of CTB differentiation (Mcmaster et al., 1995). Proteins in this cluster were predominantly mitochondrial and involved in oxidative phosphorylation. This is likely a reflection of the fact that oxygen tension plays a large role in regulating CTB invasion, and the increased abundance of proteins involved in oxidative phosphorylation is in line with the established importance of mitochondrial energetics in meeting the demands of trophoblast function. Conversely, abnormalities in mitochondrial morphology and/or function are associated with disease states such as pre-eclampsia, an example of a pregnancy complication that is associated with significant trophoblast dysfunction (Torbergsen et al., 1989).

Cluster 2 (109 proteins) consisted of proteins with a linear increase in expression from 0 h to 39 h. There was a significant enrichment in proteins with integral adhesive or migratory/invasive functions, such ESAM (Hirata et al., 2001) and CD44 (Goshen et al., 1996). Proteins in Cluster 2 were especially enriched exosomal and vacuole components. Molecules involved in autophagic vacuole formation or localization, such as LAMP-2, ATG16L1 and cathepsins S and D (Curtis et al., 2013). Autophagic activation is associated with the epithelial-to-mesenchymal transition (Gugnoni et al., 2016), a process that is one component of CTB differentiation to an invasive phenotype (DaSilva-Arnold et al., 2015; Saito and Nakashima, 2014).

Cluster 3 (102 proteins) contained proteins with a strong downregulation from 0 h to 15 h. A notable proportion of proteins (44/102) in this cluster were annotated as signal transduction molecules, including negative regulators of trophoblast migration and invasion such as endoglin (ENG) (Caniggia et al., 1997) and PAPPA2 (Winship et al., 2015).

Cluster 4 (75 proteins) contained proteins with a strong downregulation from 24 h to 39 h. Proteins in Cluster 4 showed overlap in enrichment with Cluster 3 functional processes, notably ER to Golgi transport. Although proteins in Cluster 4 did not show unique enrichment in origin, there was an enrichment in attribution to ‘acetylation’ (31/75) according to UniProtKB keywords. Interestingly, this contrasts with the upregulation of deacetylases (e.g. HDAC2) from Cluster 2. The relative activities of these enzymes remains to be determined.

### Dynamics of functionally related proteins in cultured CTBs

**Fig. 4.**
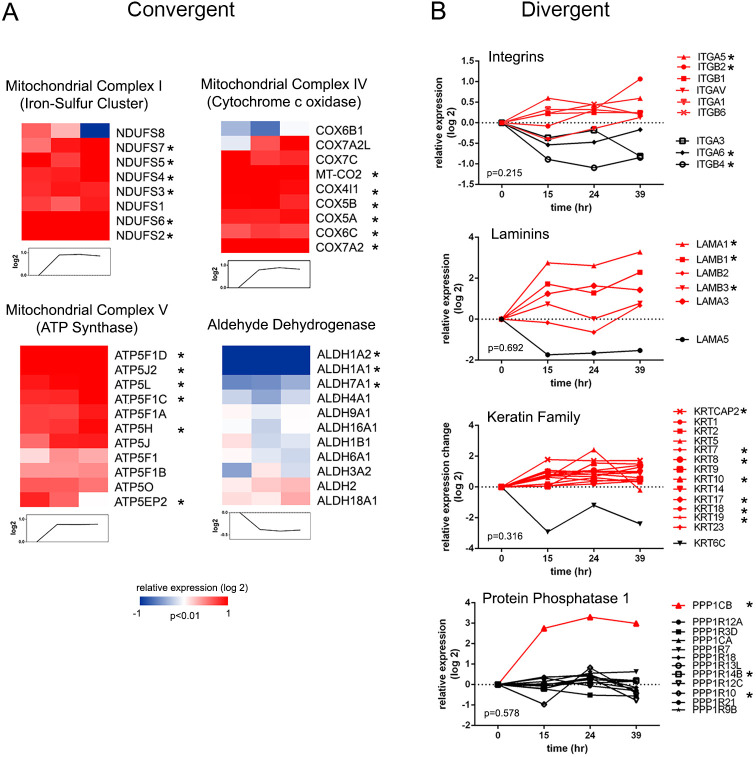
**Protein expression profiles of families/complexes during cytotrophoblast differentiation/invasion.** (A) Representative protein families/complexes with significant convergent expression profiles (*P*<0.01, distance measurement). (B) Representative protein families/complexes with significant divergent expression profiles (*P*<0.05, distance measurement). Asterisks indicate a protein that is significantly differentially expressed.

Next, we assessed the extent of temporal coordination in functionally related protein complexes and families in the proteins with significant changes in abundance over the 39 h culture period. Statistical assessment of protein clusters by annotation revealed broad coordination in expression dynamics (*P*<0.01) among protein and metabolic function subgroups. These included the iron-sulfur protein components of mitochondrial complex I, cytochrome c oxidase of mitochondrial complex IV and the ATP synthases of mitochondrial complex V (Fig. 4A), which showed significant coordinated upregulation during differentiation. Furthermore, the mitochondrial respiratory chain complex was largely captured in our analyses (Fig. S4A), broadly matching the expression profile of the energetic and metabolic processes of Cluster 1. The aldehyde dehydrogenase (ALDH) family showed coordinated downregulation matching the profile of Cluster 3, with ALDH1A1 and ALDH1A2 being the most highly differentially expressed. ALDH1A2 has previously been identified as a candidate tumor suppressor (Kim et al., 2005) and its downregulation accelerates an epithelial-to-mesenchymal transition (Seidensaal et al., 2015), an important component of CTB invasion. The annexins and cytochrome c oxidase proteins were also significantly upregulated as a group, respectively (Fig. 4B,C), underscoring previous observations of their roles in CTB function (Matsubara et al., 1997; Xu et al., 2019).

Conversely, the same statistical analysis revealed proteins that deviated from the expression profile of their corresponding complexes or families (*P*>0.05), potentially indicating a cell- or stage-specific function. The profile of the integrin subunits illustrated this phenomena. Integrin switching during CTB invasion/differentiation is a phenomenon that has been well-characterized by our group and others (Damsky et al., 1994). This was substantiated by our proteomic data, with significant upregulation of integrin α5/β1 and downregulation of α6/β4 (Fig. 4B) across culturing time. Laminins, which interact intimately with integrin ligands, are a key component of CTB extracellular matrix deposition (Blankenship et al., 1992; Yagel et al., 1988). Differential laminin distribution influences CTB function and differentiation; specifically, our data confirmed significant upregulation of the α1 and β1 chains (Korhonen and Virtanen, 1997) with time in culture. In contrast, the α5 chain tended toward downregulation, although the value did not reach significance (*P*=0.07).

A similar divergent pattern was observed with members of the serine protease inhibitor superfamily of serpins (Fig. S3D). The upregulation of SERPINE1 and SERPINB2, and the downregulation of SERPINA1 was observed in our dataset. SERPINE1 and SERPINB2, or plasminogen activator inhibitors (PAI) 1 and PAI2, are expressed by human trophoblasts *in vitro* and *in vivo* (Feinberg et al., 1989; Hofmann et al., 1994), and alterations in maternal circulation are associated with pregnancy complications with placental origins (Estellés et al., 1989; Wikström et al., 2009). SERPINA1, or α1 anti-trypsin, is an acute phase glycoprotein that may be locally synthesized and secreted by invading trophoblasts to dampen a maternal inflammatory response (Bergman et al., 1993). Although the secretome was not analyzed in this study to determine corresponding SERPINA1 release, its cellular downregulation suggests that expression may be extrinsically regulated during invasive differentiation.

Our dataset also provided insight into the expression of other protein families with divergent members. The keratins (KRT) have previously been used to profile distinct trophoblast subpopulations throughout pregnancy (Gauster et al., 2013; Mühlhauser et al., 1995; Pröll et al., 1997). Keratins were broadly represented in our dataset, with seven of the 14 detected showing significant, albeit modest, upregulation. These included KRT7, a widely used marker for villous trophoblast (Maldonado-Estrada et al., 2004). Others are associated with invasive cellular populations, e.g. KRT8, KRT17, KRT18 and KRT19. KRT6C displayed a distinct downregulated pattern; however, its associations to CTB differentiation is currently unexplored.

A unique expression pattern was observed with the serine/threonine phosphatase, protein phosphatase 1 (PPP1)’s, catalytic and regulatory subunits. Of the 12 PPP1 subunits detected, only catalytic subunit PPP1CB displayed a significant, robust upregulation. PPP1 is composed of three catalytic subunits, each with isoform-specific function and distribution (Bollen, 2001). The regulatory role of PPP1 regarding trophoblast differentiation is unclear at this time, but the expression pattern suggests that primary interactors of PPP1CB are important in this process.

### Dynamics of proteins involved in CTB invasive and migration

**Fig. 5.**
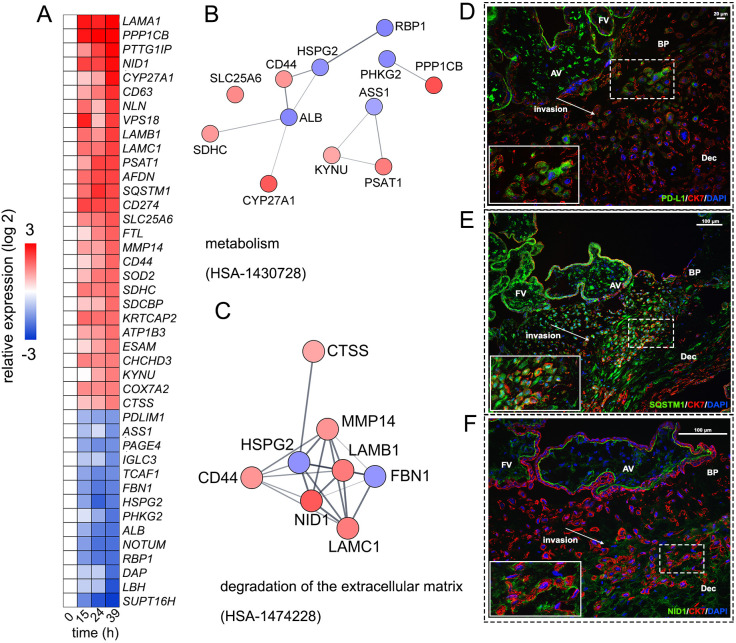
**Selected cytotrophoblast proteins with robust expression changes.** Additional cutoffs (*P*<0.01, absolute log 2 fold change ≥|1.5|) were applied to the differentially expressed (DE) proteins. (A) Heatmap depicting expression changes of 42 robust DE proteins relative to 0 h. (B,C) Predicted protein-protein interaction networks of enriched pathways. Robust DE proteins were visualized in Cytoscape using the STRING database, and molecules in significantly enriched pathways were separated to individual clusters (*P*<0.05, FDR corrected). (D-F) Representative images from second trimester tissue sections of the basal plate (BP). Sections were stained with the anti-cytotrophoblast marker cytokeratin 7 (red), with DAPI (blue) and with (D) PD-L1, (E) SQSTM1 or (F) NID1 (green). *n*≥4 biological replicates. Scale bars: 20 µm in D; 100 µm in E,F. AV, anchoring villi; BP, basal plate; FV, floating villi; Dec, decidua.

Given the number of DE proteins, we imposed additional cutoffs with the goal of visualizing the most highly robust proteins to play important roles in differentiation. Forty-two of the 477 DE proteins were stratified with more stringent criteria (*P*<0.01, absolute log 2 fold change ≥1.5 at 39 h) (Fig. 5A). STRING enrichment analysis of these proteins highlighted that robust changes occur in Reactome pathways associated with ‘metabolism’ (Fig. 5B) and ‘degradation of the extracellular matrix’ (Fig. 5C) (*P*<0.05, FDR corrected). Numerous molecules in these clusters have been associated with migratory or invasive roles, such as ASS1 (Huang et al., 2013), CD44 (Takahashi et al., 2014), CTSS (Yang et al., 2010) and MMP14 (Anacker et al., 2011). In addition, 50% of the proteins (21/42) had exosomal annotations, as determined by DAVID, highlighting the importance of their modulation during invasion.

To confirm the spatial distribution of robustly changing molecules of interest at the maternal-fetal interface, immunofluorescent staining experiments were performed on second-trimester biopsies. PD-L1, a negative regulator of T-cell signaling responsible for immune evasion (Keir et al., 2006), showed a pattern of upregulation in our proteomic data. Similar to previous observations in early and term placentas, staining patterns showed variable PD-L1 expression among CTBs, with the highest immunoreactivity occurring among the outer syncytiotrophoblast layer of villi, and more diffuse staining in subpopulations of invasive CTBs near the anchoring villi (Fig. 5D) (Veras et al., 2017).

Sequestosome 1 (SQSTM1/p62), a key regulator in the autophagic pathway (Katsuragi et al., 2015), was robustly upregulated during CTB differentiation/invasion. Autophagy-associated invasive trophoblast behavior has previously been reported to be mediated by ENG (Nakashima et al., 2013), a molecule closely associated with regulating CTB differentiation via inhibition of migratory and invasive function by TGFβ1 (Caniggia et al., 1997; Mano et al., 2011). Our proteomic data reinforce the importance of these molecules, with the concurrent upregulation of SQSTM1/p62 and the downregulation of ENG during our experimental window. DAP1, a negative regulator of autophagy, was also significantly downregulated (Koren et al., 2010). Matching previous observations in term biopsies (Nakashima et al., 2013), SQSTM1/p62 staining generally distributed in similar patterns to CK7, although CTBs deeper in the decidua exhibited weaker patterns of SQSTM1/p62 expression (Fig. 5E).

Nidogen 1 (NID1), a common component of basement membranes, also showed robust upregulation. Immunofluorescent staining of NID1 revealed a striking pattern, with nearly absent expression at the attachment site of the anchoring villi and tight spatial distribution around CTB populations deeper in the decidua (Fig. 5F). The contributions of Matrigel (Hughes et al., 2010), which are essential for the acquisition of an invasive phenotype in this model system, could not be fully discounted from our analysis. However, observations of NID1 secretion in trophoblast organoids (Turco et al., 2018) and the detection of laminin in CTB extracellular vesicles (Taylor et al., 2020), taken with the SWATH results in this study, suggest an intriguing possibility that, in select contexts, CTBs may modulate their microenvironment as part of their differentiation/invasion process. Further studies are necessary to explore this hypothesis.

### Evaluating concordance between transcript-protein

We previously published a microarray study evaluating transcriptional dynamics of CTBs differentiating towards an invasive phenotype (Robinson et al., 2017). In total, 2805 genes overlapped with the 3026 proteins (92.6%) in our dataset. The global concordance of overlapping transcript-protein pairs between samples was modest, as calculated with Spearman's correlation (average rho=0.38, Fig. S5A). This low correlation is a widely recognized phenomenon (de Sousa Abreu et al., 2009; Liu et al., 2016; Payne, 2015). The directionality in mean log2 FC from 0 h was shared by 52% and 57% of transcript-protein pairs at 15 h and 39 h, respectively (Table S2). Shared directionality improved when considering only DE transcript-protein pairs derived from the proteomic dataset, increasing to 55% and 68.4% at 15 h and 39 h, respectively. Linear associations between transcript-protein FCs from 0 h were low, with Pearson correlation coefficients of 0.13 and 0.21 at 15 h and 39 h, respectively. Correlations modestly improved when considering only DE transcript-protein pairs (r=0.27, 15 h; r=0.4, 39 h).

To determine whether differences in transcript stability explained these differences, we assessed the mRNA half-lives of transcript-protein pairs using data generated from metabolic pulse labeling (Schwanhäusser et al., 2011). mRNA half-lives of DE transcript-protein pairs were not statistically different from the matched dataset (Wilcoxon rank-sum test, Fig. S5B). Overall, these results reinforce the notion that relative transcript and protein abundances are not predictive of one another with direct comparative measures. In addition to the regulatory repertoire (such as post-transcriptional and -translational mechanisms) that impacts these types of correlations in differentiated cells with a stable phenotype, we are studying differentiating cytotrophoblasts with a dynamically changing phenotype, which further complicates these comparisons. Another factor likely to degrade correlations is our focus on primary human cells, which doubtlessly exhibit sample-to-sample variations.

As an exploratory means of identifying potential co-regulatory modules that influence CTB differentiation between the datasets, we applied consensus network analysis to the overlapping transcript-protein pairs, defining each culture time point as a trait (Langfelder and Horvath, 2007; Zhang and Horvath, 2005). This analysis identified four unique modules, two each at 0 h and 39 h, that represent expression profiles unique to those time points (*P*<0.05) (Fig. S6A,B). Gene ontology analysis revealed enriched pathways unique to these modules, with processes such as ‘embryonic development’, ‘cell-cell adhesion’ and ‘mitochondrial transport’ being negatively correlated with 0 h, or an undifferentiated state, and processes such as ‘pattern specification’, ‘focal adhesion’ and ‘RNA binding’ positively correlated with 39 h, or a differentiated/invasive state (Fig. S6C). Genes with high significance measures within these modules includes known markers of differentiation/invasion, such as ITGA5, but also molecules of demonstrated importance in murine models, such as TLE3 (Gasperowicz et al., 2013) and S100A8 (Passey et al., 1999), suggesting underexplored roles in a human cell model (Horvath and Dong, 2008) (Table S3).

## DISCUSSION

Primary cultures of villous CTBs recapitulate behaviors relevant to migration from the placental compartment, e.g. uterine invasion, and the changes in adhesive interactions that make this unusual differentiation program possible. Thus, this is a valuable model system for studying these processes in the context of pregnancy outcomes. In this study, we used SWATH-MS, a label-free data-independent acquisition method, to catalogue the global CTB proteome. This enabled a quantitative analysis of protein levels, which identified key molecules associated with CTB differentiation in culture. We discovered that 477 of the 3026 quantified proteins were differentially expressed across the time-course, with some of the most robustly changing proteins associated with ECM disassembly and migration, indicating remodeling of the CTB proteome towards an invasive phenotype. Our clustering analysis also revealed four distinct expression profiles, with notable modulation of proteins involved with oxidative phosphorylation and regulators of migratory/invasive functions. These data support the roles of numerous molecules previously identified by our group and others as regulators of CTB differentiation/invasion, and provide potential candidate molecules to be further explored in a similar context.

Three major observations emerged from our proteomic dataset. The first is the crucial role of oxidative phosphorylation in CTB differentiation/invasion. The multifaceted functions of the placenta necessitates substantial energy demands. According to GO Enrichment analysis, components of the electron transport chain (ETC) complexes were the most significantly enriched DE proteins in the global CTB proteome as a function of time in culture. Placental mitochondria have to be highly adaptable to the energy demands of their immediate environment, particularly in response to variable oxygen levels (Fisher et al., 2019). Prior to remodeling of the uterine vasculature, the placenta proper is maintained in a physiologically hypoxic environment, necessitating tight regulation of energy consumption relative to the developing fetus (Illsley et al., 2010). Although the relationship among oxygen tension, bioenergetics and CTB differentiation/invasion are important, the extent of the influence of each variable is not fully resolved. The role of oxygen in CTB differentiation is context driven (Treissman et al., 2020), with low and high levels promoting proliferation or differentiation/invasion, depending on factors such as gestational age (Caniggia et al., 2000; James et al., 2006; Wakeland et al., 2017), CTB maturation (Chang et al., 2018) and the culture system. However, the placenta is metabolically flexible, using glycolysis and oxidative phosphorylation to adapt to energy demands (Bax and Bloxam, 1997; Kolahi et al., 2017; Sferruzzi-Perri et al., 2019). Our data demonstrate that nearly all cataloged components of the ETC were upregulated in CTBs cultured under standard (20% O2) conditions, although the relevance of the downregulation of NDUFS8, a subunit of Complex I, and COX6B1, a subunit of Complex IV, is unclear. Given the role of metabolic programming in cellular differentiation, and the prevailing hypothesis that altered mitochondrial function is responsible for the pathogenesis of certain pregnancy complications, the proper modulation of ETC components during differentiation/invasion is likely to be crucial to normal pregnancy outcomes.

Second, the intriguing overlap between invasive human CTBs and cancer cells was reinforced. Invasive human CTBs use mechanisms that are implicated in the transition of benign tumor cells to a malignant phenotype (Ferretti et al., 2007). A significant difference is the tight regulation of CTB invasion, which is limited to the inner one-third of the myometrium. Thus, the dynamics of CTB expression of tumorigenic molecules often intersect those of cancer cells. For example, the divergent expression of RBP1 and SLC25A6, which is a cancer diagnostic, was observed in our dataset (Watkinson et al., 2008). Our results also demonstrate that *ALDH1A2*, a tumor suppressor gene that has a hypermethylated promoter in numerous cancers, is similarly downregulated in CTB differentiation/invasion. Meanwhile, GPRC5A, which can behave as an oncogene or tumor suppressor depending on context, was upregulated in CTBs with coincident downregulation of STAT3, suggesting that it may function as a tumor suppressor in this system (Chen et al., 2010). Another shared function between CTBs and tumor cells is the expression of immune inhibitory molecules, including HLA-G and PD-L1. Our lab has previously shown that HLA-G is restricted to CTBs differentiated along an invasive pathway (McMaster et al., 1995). Our proteomic data confirmed HLA-G expression while also observing sustained PD-L1 upregulation after culturing, affirming previous observations regarding expression of this molecule in second trimester samples (Holets et al., 2006).

Finally, our SWATH-MS experiments identified molecules whose functions are not fully known in the context of CTB differentiation, providing a valuable resource for future hypothesis generation. By assessing the global proteome of CTBs at various time points, the temporal coordination of pathways can provide insights into their functions. For example, SQSTM1/p62, a key regulator in the autophagic pathway (Katsuragi et al., 2015), was robustly upregulated during invasive CTB differentiation, along with associated components such as LC3 (MAP1LC3B) (Pankiv et al., 2007). The concurrent timing of the autophagic pathway with accompanying increases in oxidative phosphorylation and oxidative stress regulators (e.g. SOD2, GPX1) suggests further study regarding the influences of metabolic programming in CTB differentiation may be warranted (Shyh-Chang and Ng, 2017). In addition, our group has been interested in the role of histones and histone modifications in placental development. The progressive downregulation of SUPT16H, a major subunit of the FACT complex, may signal a maintenance or stabilization of chromatin structure as CTBs differentiate, with a decreased reliance on dedicated histone chaperones.

In conclusion, the compilation of the CTB global proteome at various stages of differentiation constitutes an important resource for investigators in the field that confirms previous results from our group and others, and highlights potentially fruitful areas for further mechanistic investigations of invasion. Our quantitative proteomics results give a comprehensive overview of protein abundances in time, thereby providing a unique database regarding protein expression patterns in cultured CTBs. Furthermore, given the nature of SWATH-MS, which allows continued building of a spectral library, our analyses constitute a foundation on which additional proteomic studies aimed at increasing our understanding of CTB differentiation dynamics can be constructed.

## MATERIALS AND METHODS

### Tissue collection

All methods were approved by the UCSF Institutional Review Board. Informed consent was obtained from all donors. Second trimester placentas were collected following elective terminations and placed in cytowash medium [DME/H-21 (Gibco), 12.5% fetal bovine serum (Hyclone), 1% glutamine plus (Atlanta Biologicals), 1% penicillin/streptomycin (Invitrogen) and 0.1% gentamicin (Gibco)]. Tissue samples were stored on ice prior to dissection.

### Human primary villous cytotrophoblast culture

Cytotrophoblasts (CTBs) were isolated as previously described (Hunkapiller and Fisher, 2008). CTBs intended for proteomic analyses were cultured on precoated Matrigel (BD Biosciences) plates at a density of 104,167 cells/cm^2^ in medium containing DME/H-21, 2% Nutridoma (Roche), 1% sodium pyruvate (Sigma), 1% HEPES buffer (Invitrogen), 1% GlutaminePlus (Atlanta Biologicals) and 1% penicillin/streptomycin (Invitrogen). Cells were incubated at 37°C in 5% CO_2_/95% air. Cell culture purity was determined with cytokeratin (rat polyclonal; 1:100 (Damsky et al., 1992) and vimentin (rabbit monoclonal: 1:500) immunostaining after cytocentrifugation (800 ***g***, 5 min); relative trophoblast purity above 85% was required for subsequent experimentation.

### Protein extraction and digestion

Cultured CTBs were washed with 1×PBS and detached with Trypsin (∼5 min incubation) at 15, 24 and 39 h. Cell suspensions were centrifuged at 800 ***g*** for 5 min. The supernatant was removed and cells were washed twice with 1×PBS, centrifuging after each wash. After the final wash, supernatant was removed and cell pellets were snap-frozen and stored at −80°C. To prepare samples for mass spectrometry, samples were thawed and sonicated in lysis buffer (1% SDS in 50 mM ammonium bicarbonate) and protein concentrations were quantified using Micro BCA (Thermo Scientific). Equal amounts of protein (25 μg) were reduced in 5 mM TCEP (tris carboxyethylphospine) and incubated at 60°C for 45 min. Iodoacetic acid was added to the samples at a final concentration of 10 mM and incubated at room temperature for 15 min, covered. Trypsin (Promega) was added at a 1:20 ratio (w/w) and samples were digested overnight at 37°C. Detergent was removed from the samples with Pierce Detergent Removal Columns (Thermo Scientific) according to manufacturer's protocol.

### Peptide fractionation and SWATH-MS

Peptides were separated offline into eight fractions using alkaline pH reversed-phase HPLC. Peptides from each fraction were separated using a nanoLC Ultra 2D Plus system (SCIEX) with the cHiPLC system interfaced with a 5600 Triple TOF mass spectrometer (SCIEX). The peptides were loaded onto a guard column (300 μm i.d.×5 mm, 5 μm particle size, 100 Å pore size; Acclaim PepMap300 C18; Thermo-Fisher) and were washed with aqueous phase composed of 2% solvent B [98% acetonitrile (ACN)/0.1% formic acid (FA)] in solvent A (2% ACN/0.1% FA), flow rate 12 μl/min for 3 min. The peptides were then separated on a C18 Acclaim PepMap100 column (75 μm i.d.×150 mm, 3 mm particle size, 100 Å pore size; Thermo Fisher Scientific) heated at 40°C. Peptides were eluted at a flow rate of 300 nl/min with a 90 min gradient of 2-35% solvent B. In positive-ion mode, MS scans from m/z 400-1200 were acquired followed by MS/MS scans of the 20 most abundant ions with an exclusion time of 15 s. SWATH-MS acquisition was performed with the instrumentation described above. A 50-variable-window setup for SWATH acquisitions was determined using the ‘variable window calculator’ algorithm of SCIEX. MS1 survey scans were acquired from 400-1200m/z for 250 ms and MS2 spectra were acquired in high-sensitivity mode from 100-1500m/z for 65 ms. The spectral library was constructed by processing the raw data-dependent fractionation files through ProteinPilot against a human reference proteome (UniProt). We extracted information that contains at least three transitions per peptide and three peptides per protein. Alignment of peaks was based on retention time of medium to high abundance endogenous peptides. Peak group detections were filtered at 1% FDR using the SWATH Replicates Analysis Template. Spectral libraries and SWATH-MS acquisitions were uploaded to the Illumina BaseSpace Cloud and analyses were performed in SCIEX Cloud OneOmics.

### Data availability

Raw chromatogram files have been deposited in the MassIVE (https://massive.ucsd.edu) proteomic database under accession number MSV000086970. The relative abundances of quantified proteins, with annotations, can be found in Table S1.

### Bioinformatic analysis

Normalized data were log2 transformed for downstream analysis. Gene ontology (GO) analysis was performed with DAVID (Dennis et al., 2003; Kuleshov et al., 2016) and Perseus (Tyanova and Cox, 2018) using the identified proteins in the study as the background list. Unsupervised hierarchical clustering was performed with MeV (MultiExperiment Viewer).

Transcriptomic and proteomic pairs were assessed with weighted gene co-expression analysis (WGCNA) (Zhang and Horvath, 2005). Consensus network modules were defined using a soft threshold power β=16, a minimum module size of 30 and a dynamic tree cut height of 0.65. Module-trait relationships were determined with the WGCNA package, and functional enrichment analysis of modules was determined using the WGCNA package and Enrichr (Chen et al., 2013). Modules can be found in Table S3.

### Immunofluorescent staining

Briefly, tissue was fixed in 4% paraformaldehyde and dehydrated by passing through sequential increases in sucrose before embedding in OCT (Thermo Fisher Scientific). Sections were permeabilized with methanol and rinsed with PBS before incubation in blocking buffer (5% BSA and 0.05% Tween 20). CTBs were labeled with rat anti-cytokeratin 7 (1:100, Damsky et al., 1992) with: PD-L1 (1:100, Cell Signaling, #13684), p62/SQSTM1 (1:100, Novus Biologicals, #H00008878-M01) or NID1 (1:500, R&D Systems, AF2570) for 1 h at 37°C. After three additional PBS washes, primary antibodies were detected using species-specific secondary antibodies (1:1000, Jackson ImmunoResearch Labs, see Table S4). Sections were mounted using Vectashield with DAPI (Vector Laboratories). Images were acquired using a Leica DM5000 B inverted microscope. Antibodies and their sources are described in Table S4. Tissue sections from at least four placentas were evaluated for each condition.

### Statistics

Graphs were created in R and Graphpad. Differential expression was determined using linear modeling [limma (Ritchie et al., 2015)]. Principal component analysis [FactoMineR (Lê et al., 2008)], gap statistic [NbClust (Charrad et al., 2014)], fuzzy clustering [Mfuzz (Kumar and Futschik, 2007)] and significance of protein profile similarities [proteinProfiles (Hansson et al., 2012)] were performed using their respective packages. For fuzzy clustering, relative abundance of quantified proteins was log10 transformed, Z-scored and clustered using a fuzzification value of two with a minimum membership value of 0.35. Network analysis was performed with Cytoscape using the STRING database (Shannon et al., 2003).

## Supplementary Material

Supplementary information

Reviewer comments
