# Supplementary material for: Global proteomic analyses of human cytotrophoblast differentiation/invasion
Source: Development. 2021 Jul 1;148(13):dev199561. doi: 10.1242/dev.199561 (PMC8276980; doi:10.1242/dev.199561)
Supplement: Supplementary information [file develop-148-199561-s1.pdf]

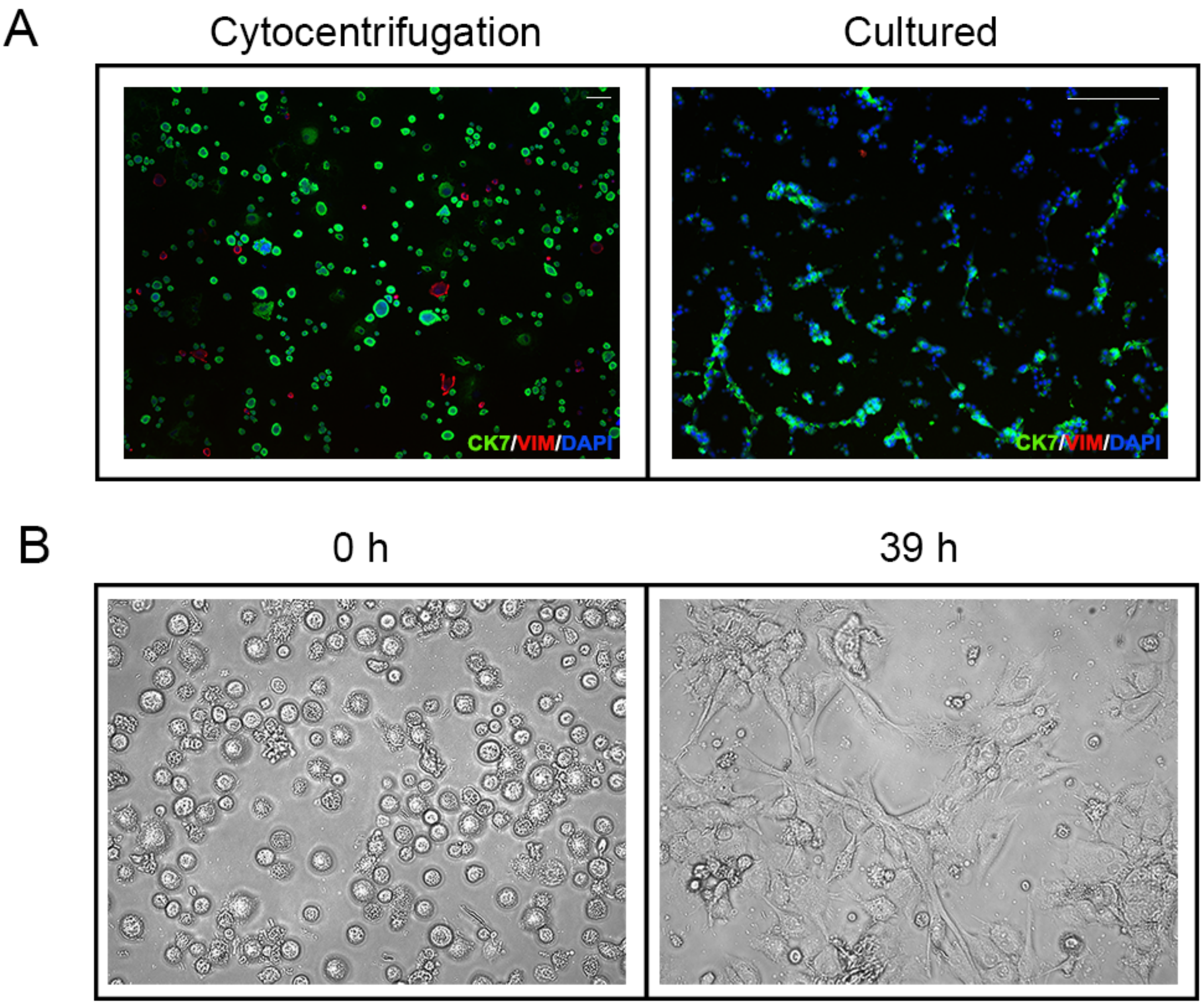

Fig S1. Primary villous cytotrophoblast (CTB) culture. (A) Representative images of culture purity prior to plating (0 h) and after plating (15 h). Cells were stained with anti-CTB marker cytokeratin (CK7, green), vimentin (VIM, red), and DAPI. (B) Representative microscopy images of CTB differentiation in culture. Scale bar = 20 or 250 μm.

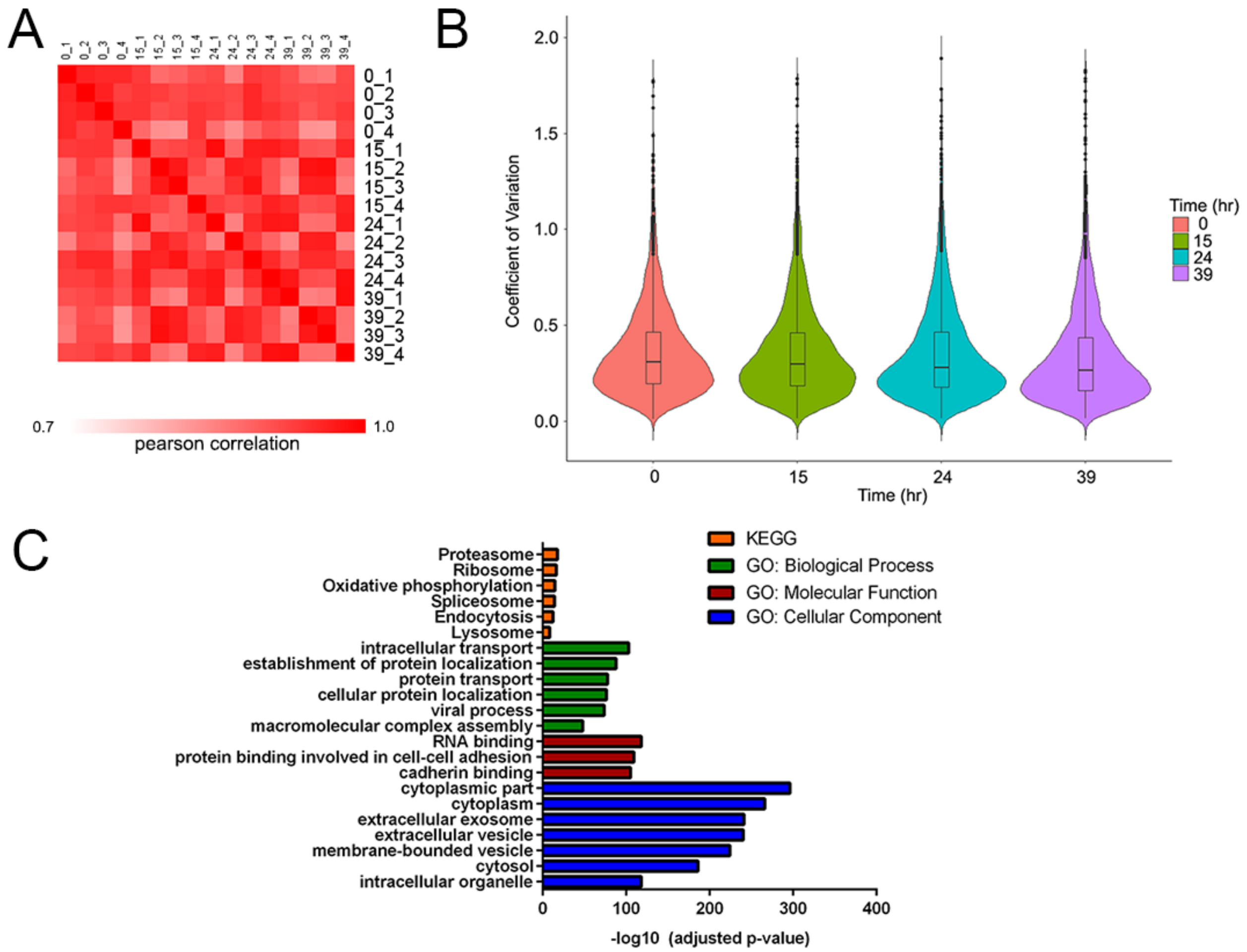

Fig S2. Features of global cytotrophoblast (CTB) proteomic dataset. (A) Heatmap of Pearson correlation scores for all biological samples used in analyses. (B) Violin plot of coefficient of error of measured proteins at each time point. (C) Gene Ontology and KEGG Pathway analysis of all quantified proteins.

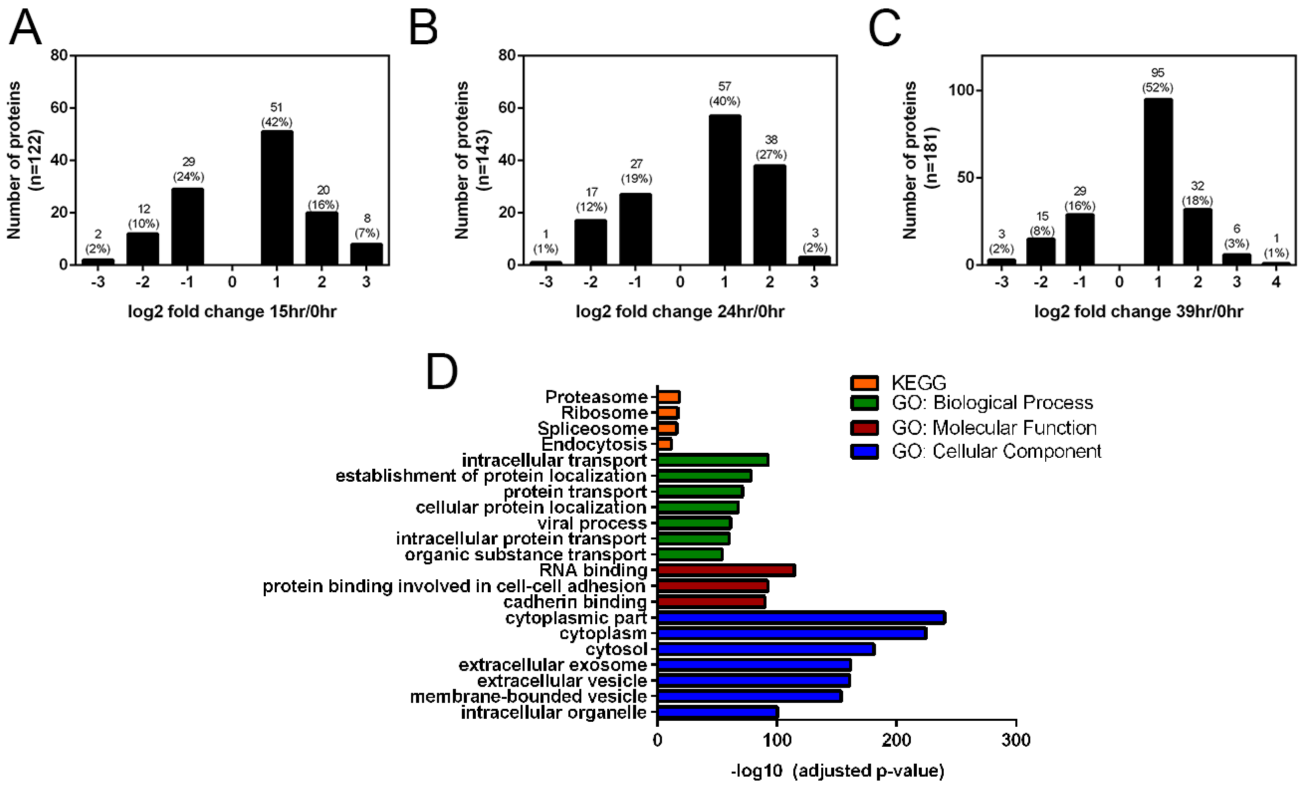

Fig S3. Features of differentially expressed (DE) proteins in global cytotrophoblast (CTB) proteomic dataset. Distribution of log fold changes in DE proteins at (A) 15 h, (B) 24 h, and (C) 39 h. (D) Gene Ontology and KEGG Pathway analysis of proteins that are not DE.

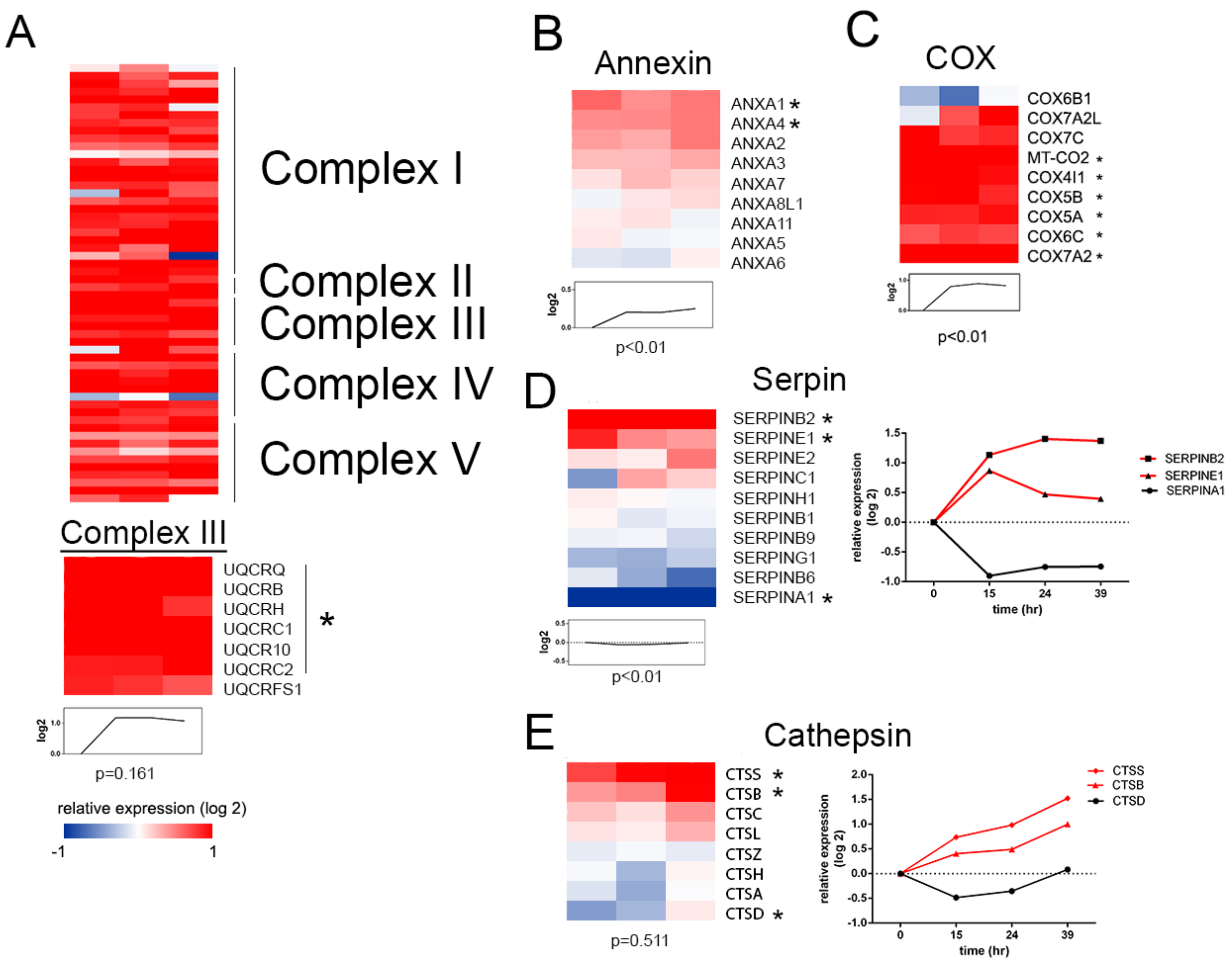

Fig S4. Protein profiles of families/complexes during cytotrophoblast (CTB) differentiation/invasion. (A) Heatmap of processes related to oxidative phosphorylation and components of the electron transport chain. (B) Heatmap of annexin proteins. (C) Heatmap of cytochrome c (COX) proteins. (D) Heatmap of serpin proteins. (E) Heatmap of cathepsin proteins. *indicates a protein that is differentially expressed.

Development • Supplementary information

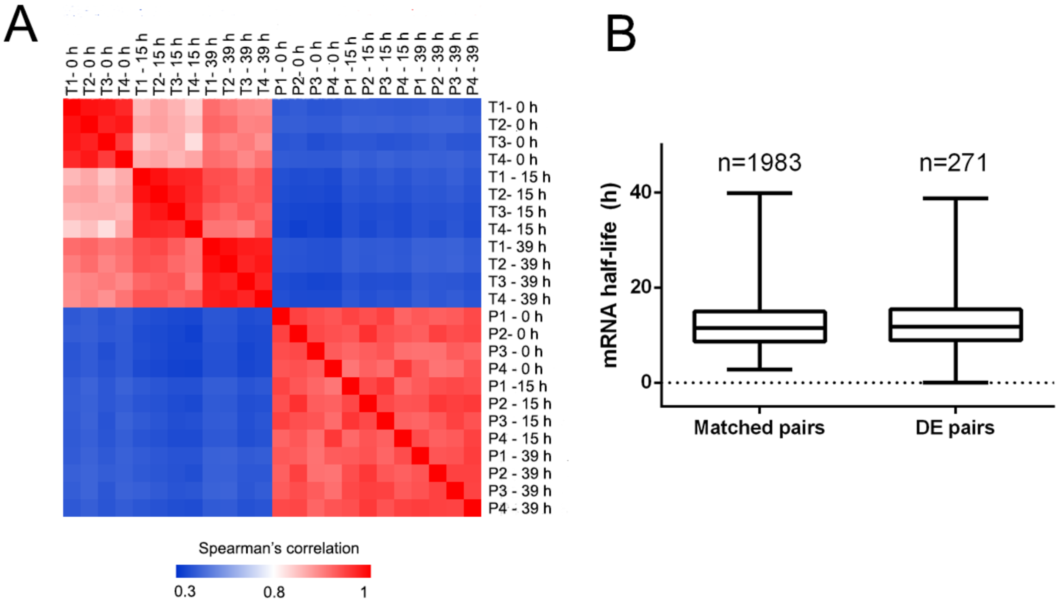

Fig S5. Transcriptome and proteome correlation analysis. (A) Heatmap of Spearman correlation coefficients of transcriptomic and proteomic samples. T= transcriptomic, P=proteomic. (B) Comparison of mRNA half-life of matched transcript-protein pairs and pairs of differentially expressed molecules derived from the proteomic dataset.

Development • Supplementary information

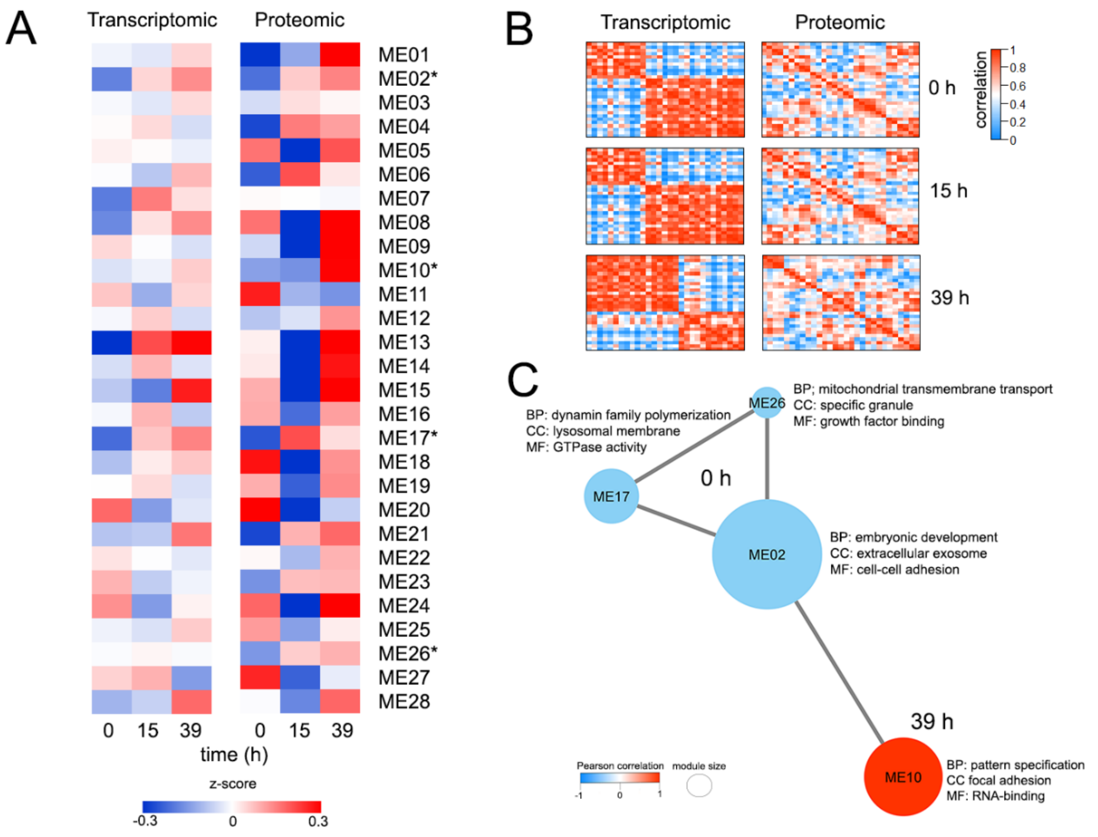

Fig S6. Transcriptome and proteome consensus network analysis. (A) Heatmap of z-scored expression values of matched transcript-protein pairs, separated into 28 modules after weighted gene cluster network analysis (WGCNA). Four modules significantly corresponded with a culture time point (*p<0.05). (B) Eigengene networks of transcriptomic and proteomic datasets at matching time points. (C) Cytotrophoblast differentiation module network. Highly enriched gene ontology terms were associated with modules negatively correlated with 0 h (blue) or positively correlated with 39 h (red).

Development • Supplementary information

Table S1. Sample information and annotated, normalized proteomic abundance data

Click here to download Table S1

Table S2. Fold change values of transcript-protein pairs

Click here to download Table S2

Table S3. Consensus network values of transcript-protein pairs

Click here to download Table S3

Table S4. List of antibody sources

Click here to download Table S4
