# Supplementary material for: Global proteomic analyses of human cytotrophoblast differentiation/invasion
Source: Development. 2021 Jul 1;148(13):dev199561. doi: 10.1242/dev.199561 (PMC8276980; doi:10.1242/dev.199561)
Supplement: Reviewer comments [file develop-148-199561_review_history.pdf]

Global proteomic analyses of human cytotrophoblast differentiation/invasion

Hao Chen, Katherine E Williams, Elaine Y Kwan, Mirhan Kapidzic, Kenisha A Puckett, Rayyan K Aburajab, Joshua F Robinson and Susan J Fisher

DOI: 10.1242/dev.199561

Editor: Liz Robertson

Review timeline

Original submission: 2 March 2021
Editorial decision: 22 March 2021
First revision received: 16 April 2021
Editorial decision: 17 May 2021
Second revision received: 25 May 2021
Accepted: 1 June 2021

Original submission

First decision letter

MS ID#: DEVELOP/2021/199561

MS TITLE: Global Proteomic Analyses of Human Cytotrophoblast Differentiation/Invasion

AUTHORS: Hao Chen, Katherine E Williams, Elaine Y. Kwan, Mirhan Kapidzic, Kenisha A Puckett, Rayyan K Aburajab, Joshua F Robinson, and Susan Fisher

I have now received all the referees' reports on the above manuscript, and have reached a decision. The referees' comments are appended below, or you can access them online: please go to BenchPress and click on the 'Manuscripts with Decisions' queue in the Author Area.

As you will see, the referees express considerable interest in your work, all agree that the data set represent a valuable resource, but have some significant criticisms and recommend a substantial revision of your manuscript before we can consider publication. If you are able to revise the manuscript along the lines suggested, which may involve further experiments, I will be happy receive a revised version of the manuscript. Your revised paper will be re-reviewed by one or more of the original referees, and acceptance of your manuscript will depend on your addressing satisfactorily the reviewers' major concerns. Please also note that Development will normally permit only one round of major revision.

We are aware that you may be experiencing disruption to the normal running of your lab that make experimental revisions challenging. If it would be helpful, we encourage you to contact us to discuss your revision in greater detail. Please send us a point-by-point response indicating where you are able to address concerns raised (either experimentally or by changes to the text) and where you will not be able to do so within the normal timeframe of a revision. We will then provide further guidance. Please also note that we are happy to extend revision timeframes as necessary.

Please attend to all of the reviewers' comments and ensure that you clearly highlight all changes made in the revised manuscript. Please avoid using 'Tracked changes' in Word files as these are lost in PDF conversion. I should be grateful if you would also provide a point-by-point response detailing

© 2021. Published by The Company of Biologists under the terms of the Creative Commons Attribution License (https://creativecommons.org/licenses/by/4.0/).

how you have dealt with the points raised by the reviewers in the 'Response to Reviewers' box. If you do not agree with any of their criticisms or suggestions please explain clearly why this is so.

Reviewer 1

Advance summary and potential significance to field

This study has investigated the global proteome of second trimester human invasive trophoblast cultured in vitro <4 days. Although understanding the genetic and phenotypic changes that occur as trophoblast invades deep into decidua is indeed an important question to address, this study has several limitations and it is not clear what the novel findings are and their relevance to in vivo.

Comments for the author

The study relies heavily on one technique and should be complemented by other experimental approaches to allow more in-depth validation. A number of issues need addressing:
1) The cells used for this study are primary cells isolated from second trimester placentas.

This raises several concerns:
a. These placental digests will contain a mixed population of villous trophoblast (VCT), a few cell columns of extravillous trophoblast (EVT) and non-trophoblast cells from the villous core as well as potential maternal contamination. The authors do mention in their methods that cell purity was determined by cytokeratin staining, however this data is not provided and there is a need to provide further evidence on the nature of the cells they have analysed as maternal epithelial contaminants also express cytokeratin. FACS analysis and/or immunofluorescence using markers for EVT, VCT and non-trophoblast cells should be performed on the cultures before proteomic studies to characterize the cell types present.
b. Trophoblast need specific culture conditions to proliferate and differentiate in 2D (Okae et al., Cell Stem Cell 2018). Without optimal conditions for trophoblast, fibroblasts that will be most likely the dominant cell type in the digest will be the ones to proliferate. Indeed, Fig S1A suggests that fibroblasts dominate the culture by 39h as this is not the typical morphology of EVT. Thus, there is a concern that the results could be reflecting the emergence of fibroblasts. Can the authors exclude their presence?
c. Invasion of EVT occurs mainly in the first trimester and the spiral arteries are mostly converted by ~18 weeks so the trophoblast isolated from these samples may well have lost the inherent invasive activity of first trimester trophoblast. Can the authors demonstrate that the cells they have isolated have retained invasive capacity?
d. When primary cells are isolated from tissues and put into culture, there are likely to be 'stress' signals during the short time window after isolation. To make sure these findings are relevant, could some of the proteins identified be validated using the human trophoblast stem cell model during their differentiation to EVT (Okae et al., 2018)?

2) Four placental samples were used for this study and it is described that the cells from these samples were pooled for proteomic analysis. This would not allow comparisons across samples to identify the statistically relevant findings.

3) Though it is reassuring that proteins that have been described in previous reports are also found as stated by the authors, for example, the integrin switch, SERPINEs and upregulation of HLA-G, it is not clear what the novel findings are. The authors should highlight these more clearly. PD-L1 has also been shown by many groups to be expressed by EVT.

4) Another way to validate these finding may be to compare these to the results of transcriptome analysis of invasive trophoblast published by several groups in the first trimester and later in pregnancy. Are the same markers upregulated in the EVT in the decidua compared to EVT emerging from the cell column?

5) The report is largely descriptive and mechanistic or functional data has not been provided to show how these proteins affect interaction with decidual cells and matrix and trophoblast

© 2021. Published by The Company of Biologists under the terms of the Creative Commons Attribution License (https://creativecommons.org/licenses/by/4.0/).

invasive behaviour. It would provide more depth to this work if some functional validation could be performed using human trophoblast stem cell lines that are now available.

Reviewer 2

Advance summary and potential significance to field

This is a solid study reporting on the identification of proteomic changes during human cytotrophoblast differentiation, starting from isolated 2nd trimester cytotrophoblast cells that are cultured in vitro for up to 39h during which they differentiate and acquire an invasive phenotype. The group has long-standing expert knowledge in this area of research. The data provide novelty as they shed light onto the proteome, and not just the much more common transcriptional changes. Bridging the gap between transcriptional profiles and how they relate to proteomic changes is an important challenge in developmental biology.

In light of this, the authors should integrate their data with existing RNA-seq data and detail the overlap they observe between mRNA and protein patterns. Conversely, exceptions to this are equally interesting, i.e. cases where protein amounts change but abundance of the respective transcripts do not, or vice versa. This is anecdotally hinted at in the text, but this should be thoroughly analyzed on primary data and displayed in a figure.

That apart, the description of the various pathways and potential links to biological processes is far to long, and should be shortended. Similarly, the current figures can be reduced/condensed to convey the key messages, without showing similar information in multiple graphs (e.g. the trajectories of expression changes).

The confirmation of novel marker proteins is minimal, and would benefit from the analysis of multiple additional staining patterns, so to enhance the benefit to the research community and novelty aspect of the study.

Overall, this study should be seen as a resource paper, and I would recommend that it should be restructured into a Short Report.

Comments for the author

Pls see above.

Reviewer 3

Advance summary and potential significance to field

Overview of study: The manuscript by Chen et al sets out to define the global proteomic landscape of human progenitor trophoblasts differentiating along the invasive extravillous cell pathway. To do this, primary cytotrophoblasts (CTB) isolated and purified from second trimester placentas were plated on Matrigel over 0, 15, 24, and 39 hour time-points and allowed to spontaneously differentiate. At each time-point, CTBs were harvested for protein. Peptides were generated, segregated by reverse LC, and subjected to quantitative proteomic analyses using a relatively new approach mass spectrometry approach called sequential window acquisition of all theoretical fragment-ion spectra (SWATH). Identified peptides were mapped to a database, protein abundances (> 3000 proteins) were determined, and from this dataset differential protein expression, kinetics and pathways were linked to stages of differentiation.

Understanding of factors that are important in progenitor trophoblast differentiation (in either the villous or extravillous pathways) is relatively limited. Most studies have used transcriptomic analyses using various in vitro models (e.g. explants, and primary CTB, and more recently human trophoblast stem cells and 3D organoids), but an unbiased approach to map the proteomic landscape during this development process has never been investigated. Therefore this is a novel study.

© 2021. Published by The Company of Biologists under the terms of the Creative Commons Attribution License (https://creativecommons.org/licenses/by/4.0/).

This is a very well written manuscript that is easy to follow and understand. The description of the findings is clear, and the interpretation and discussion of the findings in how the relate to published data as well as novel markers/pathways is excellent. This work provides an important resource to placental and developmental biologists, and as the authors indicate, also a resource for researchers in the cancer biology field. This is a descriptive and highly-focused resource, but the experimental approach used is highly appropriate and the from what I can tell, the quality of the data is high.

Comments for the author

Comments:
1) While the authors have previously published on the characterization of the primary CTB model used in the study, in the context of this study, a more complete characterization of model as a form of quality control should be performed. For instance, while morphological differences in CTB are shown in 0h and 39h of culture, it would be more complete to also present standard assessments of CTB purity, step-wise alterations in genes or proteins associated with to CTB EVT development (TEAD4, ITGA6, NOTCH1, NOTCH2, TEAD2, HLA-G, ITGA5, ITGA1 for example). While MS quantifies peptides of many of these proteins, it nonetheless verifies the model as being intact prior to feeding cells into the proteomic pipeline.

2) Related to my comment above, second trimester CTB are not usually the primary cell of choice for modeling CTB to EVT differentiation. To this end, could the authors provide a rationale or a better description for using mid-gestation CTB, rather than first trimester CTB (I realise availability to this resource is difficult)? Are the differentiation kinetics along the invasive pathway similar in CTB isolated from early or mid gestation? Would using CTB from term allow for a similar study design? These nuances in developmental timing are currently not evident, but could provide better context to the reader.

3) While only 4 biological samples were used in this study, I feel it would still nonetheless be important to include in Supplemental data any clinical characteristics of the patients that provided the placental material. While from elective terminations, some information on maternal biology (age, BMI, ethnicity) could be useful.

4) Data showing an increase in proteins associated with oxidative stress and mitochondrial function was interesting, and sheds important insight of the importance of metabolism/energy demands of EVT. However, at least in transcriptomic data, elevated or overly-represented markers linked with oxidative stress or mitochondrial function can also be readouts of cell stress and sub-optimal cell viability. Can the authors show that cell viability across the 4 time-points was intact?

Minor comments:
1) Discussion Line 334. It was not clear if the authors were referring to this current study or the study referenced at the end of the sentence.

2) The authors interpretations of the role of oxygen is not aligned with a view that low oxygen (not necessarily hypoxia) may actually be required for CTB development into intermediate/immature EVT (PMID: 28167044, PMID: 29259074, PMID: 31871275). The authors are correct in that strong data exists showing that higher levels of oxygen promote EVT invasion, but as written on Lines 332 to 338, the nuances of environmental oxygen on EVT development is not captured (and could likely be an interesting discussion point considering the manuscript is directly examining EVT differentiation).

3) Within the protein network maps (Fig 5), the text is small and very difficult to read

4) Could the authors provide magnified/digitally enlarged insets of IF images to more easily show protein localisation to specific cell types in Fig 5D, E, and F?

© 2021. Published by The Company of Biologists under the terms of the Creative Commons Attribution License (https://creativecommons.org/licenses/by/4.0/).

First revision

Author response to reviewers' comments

Response to Reviewers:

We thank the reviewers for their thoughtful and supportive comments that significantly improved the quality of the original submission. As outlined below, we have revised the manuscript to address specific concerns, suggestions, or concerns. New text in the revision is highlighted in yellow, and the location of these changes in the revised manuscript is indicated in parenthesis in our responses below.

Specific Concerns Raised by Reviewer 1:
Concern 1a. These placental digests will contain a mixed population of villous trophoblast (VCT), a few cell columns of extravillous trophoblast (EVT) and non-trophoblast cells from the villous core as well as potential maternal contamination. The authors do mention in their methods that cell purity was determined by cytokeratin staining, however this data is not provided and there is a need to provide further evidence on the nature of the cells they have analysed as maternal epithelial contaminants also express cytokeratin. FACS analysis and/or immunofluorescence using markers for EVT, VCT and non-trophoblast cells should be performed on the cultures before proteomic studies to characterize the cell types present.

Response 1a: We agree that the cell isolation method we use results in a mixture of villous and extravillous/invasive cytotrophoblasts. Work from our group over many years has shown that culturing these cells on Matrigel promotes villous cytotrophoblast differentiation into an extravillous phenotype. In the process, the original heterogenous population becomes more homogenous. This has been evident from our previous publications (Mcmaster et al. 1995) and the upregulated expression of stage-specific antigens that are modulated during this process, which include HLA-G, VEGF family members, ITGA5/β1 and MMPs. Furthermore, non-adherent cells, such as immune contaminants, are removed by washing the cultures shortly after the cytotrophoblasts adhere. Additionally, at this stage of pregnancy, we do not observe uterine epithelial cells at the maternal-fetal interface and, therefore, they do not contaminate our cultures.

To better document the nature of our cultures, we now provide an estimate of purity in the form of cytokeratin staining after cytocentrifugation and of the adherent cells, which enabled calculating the percentage of cytotrophoblasts versus other cell types (lines 452-454, Supplemental Figure 1). Finally, we agree that FACS analysis is a valuable method for characterizing many types of cells. However, application of this method to cytotrophoblasts is highly problematic due to their large size and tendency to adhere to any substrate. As a result, we have found that many cells are lost or damaged during the analysis, producing highly inconsistent data. Other groups have reported the same problem (Liu et al. 2018). That is why our data on cytotrophoblast differentiation in culture is largely based on immunolocalization, immunoblot, and RNA analyses (Zhou et al. 2013).

Concern 1b. Trophoblast need specific culture conditions to proliferate and differentiate in 2D (Okae et al., Cell Stem Cell 2018). Without optimal conditions for trophoblast, fibroblasts that will be most likely the dominant cell type in the digest will be the ones to proliferate. Indeed, Fig S1A suggests that fibroblasts dominate the culture by 39h as this is not the typical morphology of EVT. Thus, there is a concern that the results could be reflecting the emergence of fibroblasts. Can the authors exclude their presence?

Response 1b. As noted above, our cultures after washing contain cells that are greater than 95% cytokeratin-positive. Therefore, there are few fibroblast contaminants. In addition, Okae et al. did not apply their method to second trimester placentas, reasoning that the proliferative trophoblast stem cell population is much reduced. Here, our goal was to characterize the proteome of villous cytotrophoblasts differentiating to an extravillous

© 2021. Published by The Company of Biologists under the terms of the Creative Commons Attribution License (https://creativecommons.org/licenses/by/4.0/).

phenotype, rather than focusing on any stem cells that remain after the first trimester. With regard to the morphological changes noted at 39 h, the elongated shapes are characteristic of migrating and invasive cytotrophoblasts in culture and at the maternal-fetal interface.

Concern 1c. Invasion of EVT occurs mainly in the first trimester and the spiral arteries are mostly converted by ~18 weeks so the trophoblast isolated from these samples may well have lost the inherent invasive activity of first trimester trophoblast. Can the authors demonstrate that the cells they have isolated have retained invasive capacity?
Response 1c. Second trimester cytotrophoblasts retain their capacity for invasion when plated on Matrigel-coated Transwell filters, and we have documented this fact in numerous publications (Garrido-Gomez et al. 2017; Robinson et al. 2019; Zhou et al. 2013).

Concern 1d. When primary cells are isolated from tissues and put into culture, there are likely to be 'stress' signals during the short time window after isolation. To make sure these findings are relevant, could some of the proteins identified be validated using the human trophoblast stem cell model during their differentiation to EVT (Okae et al., 2018)?

Response 1d. We have an extensive publication history characterizing proteins from the primary villous trophoblast culture, both in situ and in explant models, to confirm the relevance of the system.
Poor correlations are expected in linear comparisons between transcript to protein (Liu et al. 2016), and ultimately between a stem cell and a primary cell model, which is similarly noted by the authors of the Okae et al. 2018 in their transcriptomic comparisons of primary cells and their proliferative trophoblast stem cell line. At the request of the reviewer, we performed an independent analysis of the RNA-Seq dataset of the Okae et al. cytotrophoblast-derived trophoblast stem cells (TS^{CT}) and those directed towards an extravillous trophoblast phenotype (EVT-TS^{CT}) using limma ($p \leq 0.01$, fold change $\geq |4|$ Benjamini-Hochberg correction) to generate a DE list, which is available upon request. We also performed a preliminary qualitative comparison between our protein list and the predominant RNA-Seq genes (log2 fragments per kilobase per million [FPKM] > 10) defining the Okae et al. model.
Core features of the transcriptome and proteome were similar, with 94.6% of TS^{CT} transcripts detected in our proteomic dataset. Of the predominant genes characterizing TS^{CT} and EVT- TS^{CT}, 82% were detected in our proteomic dataset, reinforcing the trophoblast nature of the cells we studied. Within Okae's dataset, 82% of the highly expressed primary EVT genes were shared with the most highly expressed EVT-TS^{CT} genes; by comparison, 80.4% EVT-TS^{CT} predominant genes were detected in our proteomic dataset.

Concern 1e. Four placental samples were used for this study and it is described that the cells from these samples were pooled for proteomic analysis. This would not allow comparisons across samples to identify the statistically relevant findings.

Response 1e. The defining feature of SWATH-MS is sample pooling for creation of a spectral library that contains proteins from all the samples. This reference map is used for subsequent identification and quantification of proteins in individual samples. Thus, individual samples were independently injected for mass spectrometric analysis to generate the data and for downstream comparative statistics. Supplemental Table 1 contains the raw data.

Concern 1f. Though it is reassuring that proteins that have been described in previous reports are also found as stated by the authors, for example, the integrin switch, SERPINEs and upregulation of HLA-G, it is not clear what the novel findings are. The authors should highlight these more clearly. PD-L1 has also been shown by many groups to be expressed by EVT.

© 2021. Published by The Company of Biologists under the terms of the Creative Commons Attribution License (https://creativecommons.org/licenses/by/4.0/).

Response 1f. We hope for the SWATH-MS spectral library to serve as a resource for hypothesis-generation, hence submitting the manuscript as a resource article. In addition to PD- L1, we described the expression dynamics of molecules unexplored in the context of CTB differentiation/invasion, such as ALDH1A2, GPRC5A, and SUPT16H.

Concern 1g. Another way to validate these finding may be to compare these to the results of transcriptome analysis of invasive trophoblast published by several groups in the first trimester and later in pregnancy. Are the same markers upregulated in the EVT in the decidua compared to EVT emerging from the cell column?

Response 1g. To address this and Reviewer 2's comments, we generated new text (lines 301- 342, 509-515), supplemental figures (Figure S5,6), and tables (Table S2,3) comparing our proteomic results with our previously published transcriptomic dataset generated from the same cell culture model of cytrophoblast differentiation/invasion.
Concern 1h. The report is largely descriptive and mechanistic or functional data has not been provided to show how these proteins affect interaction with decidual cells and matrix and trophoblast invasive behaviour. It would provide more depth to this work if some functional validation could be performed using human trophoblast stem cell lines that are now available.

Response 1h. We agree that additional experiments in the context of maternal-fetal interactions would be extremely interesting and provide increased depth to the study. However, given the challenges associated with specimen collection during the COVID epidemic, we were not able to do these additional experiments. Thus, we submitted this manuscript as a resource article.

Specific Concerns Raised by Reviewer 2

Concern 2a. In light of this, the authors should integrate their data with existing RNA-seq data and detail the overlap they observe between mRNA and protein patterns. Conversely, exceptions to this are equally interesting, i.e. cases where protein amounts change but abundance of the respective transcripts do not, or vice versa. This is anecdotally hinted at in the text, but this should be thoroughly analyzed on primary data and displayed in a figure.

Response 2a. To address this and Reviewer 1's comments, we generated new text (lines 301- 342, 509-515), supplemental figures (Figure S5,6), and tables (Table S2,3) comparing our proteomic results with our previously published transcriptomic dataset generated from the same cell culture model of cytrophoblast differentiation/invasion.

Concern 2b. That apart, the description of the various pathways and potential links to biological processes is far to long, and should be shortended. Similarly, the current figures can be reduced/condensed to convey the key messages, without showing similar information in multiple graphs (e.g. the trajectories of expression changes).
Response 2b. We respectfully request that this section of the manuscript be preserved in its original form as we think the results and related discussion will be of interest to readers.

Concern 2c. The confirmation of novel marker proteins is minimal, and would benefit from the analysis of multiple additional staining patterns, so to enhance the benefit to the research community and novelty aspect of the study.
Response 2c. Due to COVID restrictions, we have a limited ability to perform new experiments. We appreciate that this information would be valuable and intend to do these experiments once it is possible.

Concern 2d. Overall, this study should be seen as a resource paper, and I would recommend that it should be restructured into a Short Report.
Response 2d. The manuscript was originally submitted for review as a Resource and Technique paper.

© 2021. Published by The Company of Biologists under the terms of the Creative Commons Attribution License (https://creativecommons.org/licenses/by/4.0/).

Specific Concerns Raised by Reviewer 3

Concern 3a. While the authors have previously published on the characterization of the primary CTB model used in the study, in the context of this study, a more complete characterization of model as a form of quality control should be performed. For instance, while morphological differences in CTB are shown in 0h and 39h of culture, it would be more complete to also present standard assessments of CTB purity, step-wise alterations in genes or proteins associated with to CTB EVT development (TEAD4, ITGA6, NOTCH1, NOTCH2, TEAD2, HLA- G, ITGA5, ITGA1 for example). While MS quantifies peptides of many of these proteins, it nonetheless verifies the model as being intact prior to feeding cells into the proteomic pipeline.

Response 3a. The stepwise upregulation of many of the proteins noted above was recapitulated in our proteomic data (e.g., HLA-G, ITGA1, ITGA5). Please note that the question of purity was also raised by Reviewer 1. In response, we now show representative images of cytokeratin positive cells before and after plating (Supplemental Figure 1).

Concern 3b. Related to my comment above, second trimester CTB are not usually the primary cell of choice for modeling CTB to EVT differentiation. To this end, could the authors provide a rationale or a better description for using mid-gestation CTB, rather than first trimester CTB (I realise availability to this resource is difficult)? Are the differentiation kinetics along the invasive pathway similar in CTB isolated from early or mid gestation? Would using CTB from term allow for a similar study design? These nuances in developmental timing are currently not evident, but could provide better context to the reader.

Response 3b. We thank the reviewer for noting that this nuance may be unclear. As is evident from our publication record, we have studied first, second, and third trimester cytotrophoblast differentiation/invasion. Although we observe decreases in invasive capacity beginning as early as mid-first trimester, the differentiation of villous cytotrophoblasts to invasive cells is virtually identical as monitored by stage specific antigens that are modulated during this process. They include integrins, cadherins, MMPs, and many other families of molecules that we have assayed in targeted analyses. In contrast, cytotrophoblasts from term placentas tend to exhibit poor differentiation/invasion in culture.

The reason we chose to use second trimester cytotrophoblasts for this study was the fact that many more cells can be isolated from mid-gestation versus first trimester placentas. Given the protein requirements for a mass spectrometry-based analyses, using the smaller number of cells that can be isolated from first trimester was not an option.

Concern 3c. While only 4 biological samples were used in this study, I feel it would still nonetheless be important to include in Supplemental data any clinical characteristics of the patients that provided the placental material. While from elective terminations, some information on maternal biology (age, BMI, ethnicity) could be useful.

Response 3c. As these samples are from elective terminations at mid-gestation, access to the clinical characteristics of the donors, outside of drug use, was not permitted by our institutional review board that permits human subject research.

Concern 3d. Data showing an increase in proteins associated with oxidative stress and mitochondrial function was interesting, and sheds important insight of the importance of metabolism/energy demands of EVT. However, at least in transcriptomic data, elevated or overly-represented markers linked with oxidative stress or mitochondrial function can also be readouts of cell stress and sub-optimal cell viability. Can the authors show that cell viability across the 4 time-points was intact?

Response 3d. We regularly utilize cellular viability tests (lactate dehydrogenase release, Neutral Red uptake) to monitor viability of our primary cells during the culture period.

© 2021. Published by The Company of Biologists under the terms of the Creative Commons Attribution License (https://creativecommons.org/licenses/by/4.0/).

During the time interval over which our experiments were conducted, the cells do not show significant changes in viability (Robinson et al. 2019).

Concern 3e. Discussion Line 334. It was not clear if the authors were referring to this current study or the study referenced at the end of the sentence.
Response 3e. We have edited this portion of the discussion in response to concern 3f below.

Concern 3f. The authors interpretations of the role of oxygen is not aligned with a view that low oxygen (not necessarily hypoxia) may actually be required for CTB development into intermediate/immature EVT (PMID: 28167044, PMID: 29259074, PMID: 31871275). The authors are correct in that strong data exists showing that higher levels of oxygen promote EVT invasion, but as written on Lines 332 to 338, the nuances of environmental oxygen on EVT development is not captured (and could likely be an interesting discussion point considering the manuscript is directly examining EVT differentiation).

Response 3f. We thank the reviewer for pointing out our discussion did not capture the evolving nuances related to oxygen tension and trophoblast differentiation. We have edited, and introduced additional, text to the manuscript's discussion to include this important concept (lines 371-378), to which we have contributed.

Concern 3g. Within the protein network maps (Fig 5), the text is small and very difficult to read.
Response 3g. We have increased the size of the text in Figure 5.

Concern 3h. Could the authors provide magnified/digitally enlarged insets of IF images to more easily show protein localisation to specific cell types in Fig 5D, E, and F?
Response 3h. Digitally enlarged insets of the IF images have been appended to the figures.

Garrido-Gomez T, Ona K, Kapidzic M, et al. (2017) Severe pre-eclampsia is associated with alterations in cytotrophoblasts of the smooth chorion. Development 144(5):767-777 doi:10.1242/dev.146100
Liu Y, Beyer A, Aebersold R (2016) On the Dependency of Cellular Protein Levels on mRNA Abundance.
Cell 165(3):535-50 doi:10.1016/j.cell.2016.03.014
Liu Y, Fan X, Wang R, et al. (2018) Single-cell RNA-seq reveals the diversity of trophoblast subtypes and patterns of differentiation in the human placenta. Cell Res 28(8):819-832 doi:10.1038/s41422- 018-0066-y
Mcmaster MT, Librach CL, Zhou Y, et al. (1995) Human Placental Hla-G Expression Is Restricted to Differentiated Cytotrophoblasts. J Immunol 154(8):3771-3778
Robinson JF, Kapidzic M, Hamilton EG, et al. (2019) Genomic Profiling of BDE-47 Effects on Human Placental Cytotrophoblasts. Toxicol Sci 167(1):211-226 doi:10.1093/toxsci/kfy230
Zhou Y, Gormley MJ, Hunkapiller NM, et al. (2013) Reversal of gene dysregulation in cultured cytotrophoblasts reveals possible causes of preeclampsia. J Clin Invest 123(7):2862-72 doi:10.1172/jci66966

Second decision letter

MS ID#: DEVELOP/2021/199561

MS TITLE: Global Proteomic Analyses of Human Cytotrophoblast Differentiation/Invasion

AUTHORS: Hao Chen, Katherine E Williams, Elaine Y Kwan, Mirhan Kapidzic, Kenisha A Puckett, Rayyan K Aburajab, Joshua F Robinson, and Susan J Fisher

© 2021. Published by The Company of Biologists under the terms of the Creative Commons Attribution License (https://creativecommons.org/licenses/by/4.0/).

I have now received all the referees reports on the above manuscript, and have reached a decision. The referees' comments are appended below, or you can access them online: please go to BenchPress and click on the 'Manuscripts with Decisions' queue in the Author Area.

The overall evaluation is positive and we would like to publish your manuscript in Development. However you'll see that Reviewer 1 has identified some points that require clarification/additional information, so hoping you might be able to provide this with only minimal time effort. If you do not agree with any of their criticisms or suggestions explain clearly why this is so. Your manuscript will not require any further review rather I will accept it once the final version re-appears in the system.

We are aware that you may currently be unable to access the lab to undertake experimental revisions. If it would be helpful, we encourage you to contact us to discuss your revision in greater detail. Please send us a point-by-point response indicating where you are able to address concerns raised (either experimentally or by changes to the text) and where you will not be able to do so within the normal timeframe of a revision. We will then provide further guidance. Please also note that we are happy to extend revision timeframes as necessary.

<u>Reviewer 1</u>

Advance summary and potential significance to field

This study has looked at the global proteome of second trimester human villous cytotrophoblast differentiating to extravillous trophoblast in vitro in order to provide a reference dataset for the field on this critical process during the development of the placenta. Although understanding the proteomic changes that occur as trophoblast invades deep into decidua is indeed an important question to address, this study has some limitations as further evidence is needed to ensure that the dataset does indeed accurately reflect this process.

Comments for the author

The authors have addressed my concerns to some extent. However, there are still a couple of points that I feel need to be more robustly addressed, especially if this data is to serve as a resource for the field.
Reponses 1a and 1b – the cytokeratin staining in Supp Fig S1 is helpful. However, as the authors are analysing the proteome of villous cytotrophoblast differentiating to extravillous trophoblast, I think it is vital to show direct evidence that this is indeed the process being analysed and include it in this manuscript as quality control, even if it has been published before. Furthermore, in Supp Table S1 and looking at the values of HLAG for each sample across the timepoints, the increase in HLAG seems to peak at 15 h and then decreases. What is happening towards the last timepoint? Are the cells dying? Providing additional evidence on the cells being cultured and their viability would provide reassurance for others who may want to use this dataset as a reference. Please provide an additional staining for HLA-G, the extravillous trophoblast marker, and an apoptotic marker at the different stages of collection of cultures used for analysis.
Response 1d – Please make available as a supplementary data/figure the analyses performed. This would again provide further confirmation and the relevance of the findings described in this manuscript and how they also relate to the 2D in vitro model of human trophoblast (Okae et al., 2018).

<u>Reviewer 2</u>

Advance summary and potential significance to field

It is somewhat disappointing that the authors did not perform a few additional immunostainings to validate additional new candidate factors. However, they have performed the requested additional correlation analyses between RNA-seq data and these new proteomic data that has added significant value to the manuscript.

© 2021. Published by The Company of Biologists under the terms of the Creative Commons Attribution License (https://creativecommons.org/licenses/by/4.0/).

Comments for the author

It is somewhat disappointing that the authors did not perform a few additional immunostainings to validate additional new candidate factors. However, they have performed the requested additional correlation analyses between RNA-seq data and these new proteomic data that has added significant value to the manuscript.

Reviewer 3

Advance summary and potential significance to field

I thank the authors for addressing my concerns adequately. As indicated within my review of the first submission, this body of work is important as examining CTB differentiation to EVT using a proteomic lens has been a long-neglected methodology, and this manuscript provides an important resource to the community.

Comments for the author

Related to concerns that I raised during the initial submission, the authors addressed my comments well and completely. From my standpoint, I do not request any further revisions.
Additional comments:
With respect to the authors replies to reviewer 1 and 2's comments, I feel overall the revised manuscript does an adequate job in addressing them. New analyses from a transcriptomic dataset were incorporated to provide proteome-transcriptome comparisons, and I feel these were well performed and enhance the manuscript's scope and potential interest to the research community. Some responses, informed from the valid standpoint and experience of the authors, were not as carefully crafted as could have been: Response 1a. For example, in my lab's experience in isolating CTBs from first and third trimester placentas using a similar approach described by the authors, we routinely get 5% vimentin+ cell contamination in our CTB preps, that without further purification (we use EGFR-beads) result in fibroblast overrun beyond 3-4 days of culture (not the case or a major concern in this study as the culture went up to 39h). As well, we sometimes observe maternal KRT7+ contamination as evidenced from uterine gland organoid contamination in CTB organoid conditions/cultures that gradually disappear over the first few passages in CTB progenitor media. I feel some of the certainties related to cell isolation, purity, and stability in vitro that we as a field shared is gradually shifting a little now that more sophisticated tools and culture conditions have been developed that have highlighted some inconsistencies with traditional cell culture techniques.

Second revision

Author response to reviewers' comments

We thank the reviewers for their second review of our manuscript and for the helpful comments provided throughout the revision process.

Specific Concerns raised by Reviewer 1:
Concern 1a. - “The cytokeratin staining in Supp Fig S1 is helpful. However, as the authors are analysing the proteome of villous cytotrophoblast differentiating to extravillous trophoblast, I think it is vital to show direct evidence that this is indeed the process being analysed and include it in this manuscript as quality control, even if it has been published before. Furthermore, in Supp Table S1 and looking at the values of HLAG for each sample across the timepoints, the increase in HLAG seems to peak at 15 h and then decreases. What is happening towards the last timepoint? Are the cells dying? Providing additional evidence on the cells being cultured and their viability would provide reassurance for others who may want to use this dataset as a reference. Please provide an

© 2021. Published by The Company of Biologists under the terms of the Creative Commons Attribution License (https://creativecommons.org/licenses/by/4.0/).

additional staining for HLA-G, the extravillous trophoblast marker, and an apoptotic marker at the different stages of collection of cultures used for analysis."

Response 1a. The noted decrease of HLA-G expression from 15 h to 39 h is statistically nonsignificant (limma, p=0.16). We have previously published immunofluorescence quantification of HLA-G expression in cultured primary human cytotrophoblasts across gestational ages (Mcmaster et al. 1995) with similar levels of experimental error. Concerns regarding culture viability were also expressed by Reviewer 3 (Concern 3d) in the first review of the manuscript. In our experience, primary human cytotrophoblasts do not undergo significant levels of cytotoxicity within the tested timeframe. We regularly utilize cellular viability tests (lactate dehydrogenase release, Neutral Red uptake) to monitor viability of our primary cells during the culture period. During the time interval over which our experiments were conducted, the cells do not show significant changes in viability (Robinson et al. 2019). In addition, classic apoptotic markers (e.g. BAD, CASP3, FADD, BAX) do not show significant increases in relative expression within our proteomic dataset (Table S1).

Concern 1b. – Please make available as a supplementary data/figure the analyses performed. This would again provide further confirmation and the relevance of the findings described in this manuscript and how they also relate to the 2D in vitro model of human trophoblast (Okae et al., 2018).

Response 1b. As noted in our previous response, we would be happy to provide the proteomic-transcriptomic comparative analysis for the individual reviewer. Furthermore we agree with Reviewer 1, and others, regarding the importance of analyses comparing the cells utilizing the Okae protocol and primary and stem cell protocols utilized in our laboratory. We are currently performing RNA-Seq analyses of these three cell models for direct transcriptomic comparison, and this data will be made publicly available upon completion.

Specific Concerns raised by Reviewer 2:
Concern 2a. It is somewhat disappointing that the authors did not perform a few additional immunostainings to validate additional new candidate factors.

Response 2a. We share the reviewer's desire for validation of new candidate factors and hope to proceed with these experiments as soon as possible.

Specific Concerns raised by Reviewer 3:
Concern 3a. Some responses, informed from the valid standpoint and experience of the authors, were not as carefully crafted as could have been: Response 1a. For example, in my lab's experience in isolating CTBs from first and third trimester placentas using a similar approach described by the authors, we routinely get 5% vimentin+ cell contamination in our CTB preps, that without further purification (we use EGFR-beads) result in fibroblast overrun beyond 3-4 days of culture (not the case or a major concern in this study as the culture went up to 39h)

Response 3a. We agree with the reviewer and note similar experimental observations regarding the percent of vimentin+ cell contamination (5-15%) immediately pre-plating, as well as fibroblast expansion beyond 4 days in culture, without additional purification methodologies. However, in addition to the mentioned methodologies (e.g. EGFR-beads, or CD45+ magnetic beads), in our experience we found that vimientin+ cell contamination after plating is significantly lowered (<5%) after wash and thus agree with the reviewer that the contributions of any contaminating cells are minimal to the dataset in the timeframe tested.

Concern 3b. I feel some of the certainties related to cell isolation, purity, and stability in vitro that we as a field shared is gradually shifting a little now that more sophisticated tools and culture conditions have been developed that have highlighted some inconsistencies with traditional cell culture techniques.

Response 3b. We agree with the reviewer and appreciate the valuable input and discussion.

Mcmaster MT, Librach CL, Zhou Y, et al. (1995) Human Placental Hla-G Expression Is Restricted to Differentiated Cytotrophoblasts. J Immunol 154(8):3771-3778

© 2021. Published by The Company of Biologists under the terms of the Creative Commons Attribution License (https://creativecommons.org/licenses/by/4.0/).

Robinson JF, Kapidzic M, Hamilton EG, et al. (2019) Genomic Profiling of BDE-47 Effects on Human Placental Cytotrophoblasts. Toxicol Sci 167(1):211-226 doi:10.1093/toxsci/kfy230

Third decision letter

MS ID#: DEVELOP/2021/199561

MS TITLE: Global Proteomic Analyses of Human Cytotrophoblast Differentiation/Invasion

AUTHORS: Hao Chen, Katherine E Williams, Elaine Y Kwan, Mirhan Kapidzic, Kenisha A Puckett, Rayyan K Aburajab, Joshua F Robinson, and Susan J Fisher
ARTICLE TYPE: Techniques and Resources Article

I am happy to tell you that your manuscript has been accepted for publication in Development, pending our standard ethics checks.

© 2021. Published by The Company of Biologists under the terms of the Creative Commons Attribution License (https://creativecommons.org/licenses/by/4.0/).
